# Prolonged hyperlipidemia exposure increases the risk of arterial stiffness in young adults: a cross-sectional study in a cohort of Chinese

**DOI:** 10.1186/s12889-020-09211-5

**Published:** 2020-07-11

**Authors:** Haojia Chen, Youren Chen, Weiqiang Wu, Zekai Chen, Zefeng Cai, Zhichao Chen, Xiuzhu Yan, Shouling Wu

**Affiliations:** 1 Shantou University Medical College, NO.22 Xinling Road, Jinping District, Shantou City, 515041 Guangdong Province China; 2 Department of Cardiology, First Hospital of Medical College of Shantou University, NO.57 Changping Road, Jinping District, Shantou City, 515041 Guangdong Province China; 3 Department of Cardiology, Second Affiliated Hospital of Shantou University Medical College, No. 69 Dongxia North Road, Jinping District, Shantou City, 515041 Guangdong Province China; 4 School of Foreign Language, Guangdong Polytechnic Normal University, NO.293 Zhongshan Avenue West, Tianhe District, Guangzhou, 510665 Guangdong China; 5 Department of Cardiology, Kailuan Hospital, North China University of Science and Technology, NO.57 Xinhua East Road, Lubei District, Tangshan City, 063001 Hebei Province China

**Keywords:** Hyperlipidemia, Arterial stiffness, Risk factor

## Abstract

**Background:**

Hyperlipidemia is associated with arterial stiffness. Herein, We examined the effect of prolonged exposure to hyperlipidemia on the risk of arterial stiffness in young adults.

**Methods:**

A study cohort (35–55 years old) that received health check-ups in the Kailuan study (2014–2016) were assessed. Hyperlipidemia was defined as a low-density lipoprotein cholesterol ≥160 mg/dL according to the Chinese Guidelines for the Management of Dyslipidemia in Adults. Subjects were divided into three groups based on the number of years with hyperlipidemia: normal (0 years), low exposure (1–5 years), and high exposure (5–10 years) groups. Arterial stiffness was defined as brachial-ankle pulse wave velocity > 1400 cm/s. For all subjects and subjects that did not meet statin treatment criteria under guidelines, logistics regression was used to analyze the effect of prolonged hyperlipidemia exposure on arterial stiffness in different age groups.

**Results:**

Among 12,431 subjects, the mean age was 46.42 ± 5.34 years with 9000 men (72.4%). Brachial-ankle pulse wave velocity gradually increased with increased exposure duration. Logistic regression analysis showed that hyperlipidemia exposure was a risk factor for arterial stiffness in the low (1.22 times) and high (1.49 times) exposure groups compared with the normal group. In the different age groups, the risk of arterial stiffness increased with the duration of hyperlipidemia exposure, apart for the 35–40-year-old population. The effect of hyperlipidemia exposure duration on arterial stiffness in young adults that did not meet statin treatment criteria under guidelines was similar to the general population.

**Conclusions:**

Prolonged exposure to hyperlipidemia in young adults increases the risk of arterial stiffness. Young adults with this condition may benefit from more aggressive primary prevention.

**Trial registration:**

Name of the registry: Risk factors and intervention for cardiology, cerebrovascular and related disease (Kailuan Study)

Trial registration number: CHiCTR-TNC1100 1489

Date of registration: Aug 24, 2011

URL of trial registry record: http://www.chictr.org.cn/showproj.aspx?proj=8050

## Background

Over the past three decades, there has been a global gradual increase in blood lipid levels, following the rising prevalence of hyperlipidemia, particularly in young adults. According to data from the National Health and Nutrition Examination Survey, 11.7 and 41.2% of adults aged 20–39 and 40–64 years old, respectively, have elevated low-density lipoprotein cholesterol (LDL-C) levels [1]. Studies have shown that dyslipidemia with elevated LDL-C is a major risk factor for cardiocerebrovascular events [2–6]. Current guidelines recommend that medical treatment of dyslipidemia should be based on LDL-C levels, along with diabetes, and 10-year risk for cardiovascular disease [7, 8]; as a result, relatively fewer younger adults meet statin recommendation thresholds. However, previous studies have demonstrated that early lipid intervention can reduce the occurrence of cardiocerebrovascular events in young adults [9, 10]. As shown in a randomized trial [9] in an adult population with an LDL-C of ≥190 mg/dL, the risk of coronary heart disease was reduced by 28% in the drug-treated group compared with the control group.

The development of cardiocerebrovascular events takes decades. Arterial stiffness begins earlier in life, and occurs early than its clinical manifestations, including the occurrence of cardiocerebrovascular events. Indeed, arterial stiffness is a risk factor for cardiocerebrovascular events [11–13]. Furthermore, hyperlipidemia, as well as being a risk factor for cardiocerebrovascular events, is associated with arterial stiffness [14, 15]. Thus, studies examining the impact of prolonged LDL-C exposure on the risk of arterial stiffness in young adults may provide a theoretical basis for delaying the progression of arterial stiffness, and even cardiocerebrovascular events, in young adults. However, to our knowledge there are no relevant studies in this area. Thus, in the present study we analyzed the effect of prolonged hyperlipidemia exposure on the risk of arterial stiffness in young adults aged 35–55 years based on data from the Kailuan study (registration number: CHiCTR-TNC-1100 1489).

## Methods

### Study participants

The Kailuan study [16–18] was performed from 2006 to 2008, and involved health check-ups (including anthropometric measurements and biochemical tests) for the in-service and retired employees of Kailuan Group in 11 hospitals, including Kailuan General Hospital and Kailuan Hospital Branch. The medical staff who participated in the first session of health check-ups performed a total of five physical examination sessions (once every 2 years) for the same population at the same site using the same protocol (including the survey content, physical examinations, and laboratory tests). In 2014, brachial-ankle pulse wave velocity (BaPWV) was randomly measured in the observed population. The study was conducted in accordance with the ethical guidelines of the 1975 Declaration of Helsinki, and the protocol was approved by Kailuan General Hospital. All subjects signed the informed consent forms.

The inclusion criteria included: i) in-service and retired employees of Kailuan Group who participated in the health check-ups between 2006 and 2014; ii) 35–55 years old when taking the health check-ups in the period of 2014–2016; and iii) agreed to participate in this study and signed the informed consent forms. The exclusion criteria were: i) without age and BaPWV data; ii) a history of cardiocerebrovascular events or tumors; iii) without data on LDL-C in 2014 and 2016; iv) without LDL-C data from any two of four sessions from 2006 to 2014; and v) with an ankle-brachial index < 0.9.

### Data collection

The epidemiological surveys, anthropometric measurements, and biochemical tests were previously reported [16, 19, 20]. Before blood pressure (BP) measurement, subjects were asked not to smoke or drink tea or coffee within 30 min, and were requested to rest in a chair for 15 min. The right radial artery BP was measured using a calibrated mercury sphygmomanometer. Systolic blood pressure (SBP) was read at the first appearance of Korotkoff sounds.

BaPWV was measured using an arteriosclerosis detection device (Omron Health & Medical (China) Co., Ltd., BP-203 RPE III). The temperature of the examination room was kept at 22–25 °C. Subjects were asked not to smoke or drink tea or coffee before the measurement. After each subject rested for at least 5 min, the basic data including gender, age, height, and weight were recorded. Subjects were then asked to wear a thin coat. At the beginning of the measurement, subjects were kept quiet, the pillow was removed, and the subject lay in a prone position. Both hands were placed on both sides of the body, with palms upwards. The BP cuffs of the four limbs were tied to the upper arms and the ankles. The cuffs were placed over the bare upper arms with the artery mark positioned directly over the brachial artery, and the lower edge of the cuff was approximately 2–3 cm away from the antecubital fossa. The marker of the lower limb cuff was located on the inner side of the lower limb, with the lower edge of the cuff approximately 1–2 cm away from the medial malleolus. The heart sound collection device was placed in the precordial region of the subject, and the left and right wrists were connected with clip leads. The measurements were performed twice for each subject, and the second measurement data were used as the final result. According to the report released by the American College of Cardiology in 1993, the normal reference range for BaPWV is < 1400 cm/s, and a BaPWV > 1400 cm/s indicates arterial stiffness. And a certain level of arterial stiffness is associated with arteriosclerosis. In the present study, BaPWV was measured on both the left and right sides, and the highest measured value was used for analysis.

### Biochemical measurements

The subjects were fasted for at least 8 h, and 5 ml of fasting venous blood taken from the antecubital fossa was collected into EDTA vacuum tubes at 7–9 AM on the day of the physical examination. After centrifugation at room temperature (24 °C) at 3000 revolutions per min for 10 min, the upper layer was harvested for detection within 4 h. LDL-C was measured with an enzymatic method (Mind Bioengineering Co. Ltd., Shanghai, China), and fasting blood glucose was measured by the hexokinase method (7600 Auto matic Analyzer, Hitachi, Tokyo, Japan). All biochemical variables were measured by an automatic biochemical analyzer (Hitachi 747; Hitachi, Tokyo, Japan).

### Relevant definitions

The diagnosis of diabetes was made if the subject was currently taking hypoglycemic drugs or had a fasting blood glucose level of ≥7.0 mmol/L. A diagnosis of hypertension was made if one of the following criteria was met: i) a previous history of hypertension; or ii) SBP ≥140 mmHg (1 mmHg = 0.133 Kpa) and/or diastolic BP ≥90 mmHg; or iii) current user of antihypertensive drugs despite an SBP of < 140 mmHg or a diastolic BP of < 90 mmHg.

Hyperlipidemia was diagnosed according to the Chinese Guidelines for the Management of Dyslipidemia in Adults [8], which recommends the use of LDL-C as an indicator for assessing the risk of cardiocerebrovascular events. Therefore, we used a definition of hyperlipidemia based on the LDL-C level (elevated if LDL-C ≥ 160 mg/dL). Because the health check-ups were arranged every 2 years in the Kailuan study, we used the blood lipid level at the time of health check-ups as the blood lipid level of the observed population within 2 years. For subjects with missing blood lipid data, we used the previous blood lipid measurement results. Because the Kailuan study began in 2006, we were unable to track the lipid levels of the observed population before the study, and we assumed that the observed population had not been exposed to hyperlipidemia before the study was performed.

### Statistical methods

Physical examination data were entered by the assigned personnel and summarized by the Kailuan General Hospital. Statistical analysis was performed with statistical software (SAS 9.4: v9.4; SAS Institute, Inc., Cary, NC, USA). Continuous variables were presented as mean ± standard deviation, and multiple comparisons were performed by one-way analysis of variance. During the pairwise comparisons of means, the LSD test was performed for equal variances, while the Dunnett’s T3 test was performed for unequal variances. Categorical variables were presented as n (%), and inter-group comparisons were based on the chi square test. Logistics regression was used to analyze the effect of the duration of hyperlipidemia exposure on arterial stiffness in different age groups. For subjects that did not meet statin treatment criteria under guidelines, logistics regression was again used to analyze the effect of hyperlipidemia on arterial stiffness in the different age groups. In Logistics regression, the duration of hyperlipidemia exposure was taken as the dependent variable, arterial stiffness as the independent variable, and the normal group as the control group, in which age, gender, SBP, high-density lipoprotein cholesterol, waist circumference, physical exercise, anti-hypertensive treatment, smoking, alcohol consumption, diabetes and baseline LDL-C were adjusted. A *P* value of < 0.05 was considered statistically significant. All analyses were two-sided test. Because both hypertension and diabetes can affect arterial stiffness, hypertensive and diabetic patients were separately ruled out in the sensitivity analysis.

## Results

### Study subjects’ general information

**Fig. 1:**
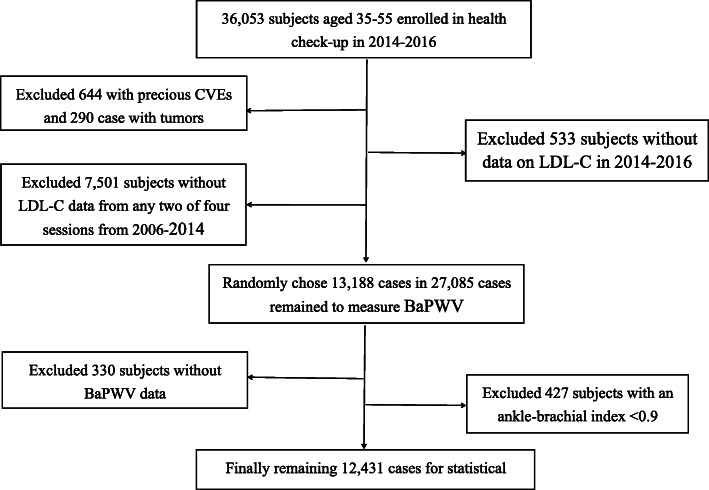
Inclusion/exclusion flowchart for study subjects

The total number of observed subjects aged 35–55 years during the 2014–2016 period was 36,053, of whom 644 were excluded because of cardiocerebrovascular events, 290 because of tumors, 533 because of missing LDL-C data in 2014–2016, and 7501 because of missing LDL-C data in any two of four sessions from 2006 to 2014. Of the 27,085 remaining subjects, 13,188 received BaPWV measurement, of whom 330 were ruled out because of missing BaPWV data and 427 because of a low ankle-brachial index (< 0.9). A final 12,431 subjects were included in our analysis, with 9000 men (72.4%) and 3431 women (27.6%), and with an average age of 46.42 ± 5.34 years (Fig. 1).

Subjects were divided into three groups according to the duration of exposure to hyperlipidemia: normal group (< 55 years old and without hyperlipidemia), low exposure group (1–5 years of hyperlipidemia exposure), and high exposure group (5–10 years of hyperlipidemia exposure). The means or proportions of age, waist circumference, body mass index, total cholesterol, high-density lipoprotein cholesterol, LDL-C, SBP, proportion of men, alcohol consumption, physical exercise, smoking, diabetes, hypertension, and use of antihypertensive drugs increased with exposure duration (*P* < 0.05), as did the mean BaPWV value and the proportion of arterial stiffness (*P* < 0.05) (Table 1).

**Table 1:** Characteristics of Participants Stratified by Years of Hyperlipidemia

variable	Normal (*N* = 4202)	Low exposure (*N* = 4485)	High exposure (*N* = 3744)	Total (*N* = 12,431)	*P different*
Age, year	45.36 ± 5.46	46.52 ± 5.35	47.48 ± 4.97	46.42 ± 5.34	< 0.01
Men, n (%)	2742 (65.3)	3265 (72.8)	2993 (79.9)	9000 (72.4)	< 0.01
Waist circumference, cm	84.96 ± 9.67	86.54 ± 9.62	87.70 ± 9.38	86.36 ± 9.63	< 0.01
Body mass index, kg/m^2^	24.57 ± 3.39	25.19 ± 3.34	25.56 ± 3.29	25.09 ± 3.37	< 0.01
Total cholesterol, mg/dl	4.37 ± 0.54	5.23 ± 1.36	6.08 ± 1.04	5.19 ± 1.25	< 0.01
HDL, mg/dl	1.31 ± 0.59	1.37 ± 0.62	1.41 ± 0.53	1.36 ± 0.59	< 0.01
LDL-C, mg/dl	2.30 ± 0.54	2.89 ± 1.59	3.29 ± 0.88	2.81 ± 1.18	< 0.01
SBP, mm Hg	127.2 ± 16.39	130.6 ± 16.38	133.9 ± 16.84	130.5 ± 16.73	< 0.01
BaPWV, cm/s	1430 ± 257	1492 ± 280	1550 ± 293	1489 ± 281	< 0.01
Anti-hypertesion, n (%)	339 (8.1)	460 (10.3)	605 (16.2)	1404 (11.3)	< 0.01
Physical exercise, n (%)	257 (6.4)	288 (6.8)	296 (8.2)	841 (7.1)	< 0.01
Hypertension, n (%)	1035 (24.6)	1456 (32.5)	1497 (40.0)	3988 (32.1)	< 0.01
Diabetes, n (%)	460 (10.9)	752 (16.8)	822 (22.0)	2034 (16.4)	< 0.01
Alcohol consumption, n (%)	2743 (65.3)	2668 (59.5)	1871 (50.0)	7282 (58.6)	< 0.01
Smoking, n (%)	1462 (34.8)	1852 (41.3)	1712 (45.7)	5026 (40.4)	< 0.01
Arterial stiffness, n (%)	1986 (47.3)	2567 (57.2)	2475 (66.1)	7028 (56.5)	< 0.01

*HDL* high-density lipoprotein cholesterol, *LDL-C* low-density lipoprotein cholesterol, *SBP* systolic blood pressure, *BaPWV* brachial-ankle pulse wave velocity

### Logistic regression analysis of hyperlipidemia exposure and BaPWV in different age groups

After adjusting for other confounding factors, the Odds Ratios (95% confidence intervals) of arterial stiffness in the low exposure and high exposure groups were 1.22 (1.08–1.37) times and 1.49 (1.30–1.72) times, respectively, that of the normal group. The low exposure group was not statistically different from the normal group in the different age groups. However, in the high exposure group the Odds Ratios of arterial stiffness were 1.47, 1.38, and 1.30 times those of the normal group in the 40–45, 45–50, and 50–55-year-old population, respectively; no differences were seen in the 35–40-year-old group. The 40–45, 45–50, and 50–55-year-old populations had a significantly increased risk of arterial stiffness with the duration of hyperlipidemia exposure (*P* trend < 0.05) (Table 2). The effect of the duration of hyperlipidemia exposure on arterial stiffness showed no significant change in young adults that did not meet statin treatment criteria under guidelines compared with the general population (Table 3).

**Table 2:** The effects of hyperlipidemia on arterial stiffness

Group		Model 1 OR (95% *CI*)	*P*	*P* trend	Model 2 OR (95% *CI*)	*P*	*P* trend
Age (year)	variable						
[35–55]	0	1(Ref)			1(Ref)		
	[1–5)	1.42 (1.30–1.56)	< 0.01		1.22 (1.08–1.37)	< 0.01	
	[5–10]	2.03 (1.84–2.25)	< 0.01	< 0.01	1.49 (1.30–1.72)	< 0.01	< 0.01
[35–40)	0	1(Ref)			1(Ref)		
	[1–5)	1.64 (1.34–2.02)	< 0.01		1.15 (0.89–1.50)	0.28	
	[5–10]	2.10 (1.65–2.67)	< 0.01	< 0.01	1.18 (0.85–1.50)	0.33	0.28
[40–45)	0	1(Ref)			1(Ref)		
	[1–5)	1.61 (1.33–1.94)	< 0.01		1.20 (0.98–1.56)	0.06	
	[5–10]	2.43 (1.99–2.97)	< 0.01	< 0.01	1.47 (1.11–1.94)	< 0.01	< 0.01
[45–50)	0	1(Ref)			1(Ref)		
	[1–5)	1.30 (1.10–1.52)	< 0.01		1.18 (0.98–1.43)	0.09	
	[5–10]	1.80 (1.52–2.12)	< 0.01	< 0.01	1.38 (1.11–1.73)	< 0.01	< 0.01
[50–55]	0	1(Ref)			1(Ref)		
	[1–5)	1.11 (0.93–1.31)	0.25		1.04 (0.84–1.28)	0.73	
	[5–10]	1.44 (1.21–1.72)	< 0.01	< 0.01	1.30 (1.03–1.64)	0.03	0.03

Model 1 unadjust;

Model 2 adjust age, gender, systolic blood pressure, high-density lipoprotein cholesterol, waist circumference, physical exercise, anti-hypertensive treatment, smoking, alcohol consumption, diabetes, baseline low-density lipoprotein cholesterol;

**Table 3:** The effects of hyperlipidemia on arterial stiffness without recommended for statin therapy at baseline^a^

Group		Model 1 OR (95% *CI*)	*P*	*P* trend	Model 2 OR (95% *CI*)	*P*	*P* trend
Age (year)	variable						
[35–55]	0	1(Ref)			1(Ref)		
	[1–5)	1.42 (1.30–1.56)	< 0.01		1.16 (1.03–1.30)	< 0.01	
	[5–10]	2.03 (1.84–2.25)	< 0.01	< 0.01	1.38 (1.20–1.59)	< 0.01	< 0.01
[35–40)	0	1(Ref)			1(Ref)		
	[1–5)	1.63 (1.32–2.00)	< 0.01		1.18 (0.91–1.54)	0.23	
	[5–10]	1.90 (1.48–2.44)	< 0.01	< 0.01	1.18 (0.84–1.65)	0.36	0.30
[40–45)	0	1(Ref)			1(Ref)		
	[1–5)	1.51 (1.24–1.84)	< 0.01		1.25 (0.99–1.65)	0.05	
	[5–10]	2.23 (1.79–2.78)	< 0.01	< 0.01	1.52 (1.13–2.06)	< 0.01	< 0.01
[45–50)	0	1(Ref)			1(Ref)		
	[1–5)	1.26 (1.07–1.50)	0.01		1.14 (0.93–1.41)	0.26	
	[5–10]	1.72 (1.43–2.07)	< 0.01	< 0.01	1.37 (1.07–1.76)	0.02	0.02
[50–55]	0	1(Ref)			1(Ref)		
	[1–5)	1.07 (0.85–1.22)	0.86		1.07 (0.84–1.34)	0.66	
	[5–10]	1.38 (1.34–1.68)	< 0.01	< 0.01	1.38 (1.05–1.81)	0.03	0.02

Model 1 unadjust;

Model 2 adjust age, gender, systolic blood pressure, high-density lipoprotein cholesterol, waist circumference, physical exercise, anti-hypertensive treatment, smoking, alcohol consumption, diabetes, baseline low-density lipoprotein cholesterol;

^a^low-density lipoprotein cholesterol ≥4.9 or total cholesterol ≥7.2; diabetes and 1.8 ≤ low-density lipoprotein cholesterol < 4.9 or 3.1 ≤ total cholesterol< 7.2 and age ≥ 40

### Sensitivity analysis

A sensitivity analysis was performed after diabetic and hypertensive patients were separately excluded. After adjusting for the confounding factors, the relationship between hyperlipidemia exposure and arterial stiffness in the different age groups did not change after exclusion of the diabetic patients. After exclusion of the hypertensive patients, the relationship between hyperlipidemia exposure and arterial stiffness did not change in the 35–55-year-old population. Among the different age groups, only the 45–50- and 50–55-year-old populations had significantly higher Odds Ratios in the high exposure group (increased by 1.45 and 1.49 times, respectively) compared with the normal group (Table 4).

**Table 4:** Logistics analysis after excluding person with diabetes or hypertension

Group		Excluding diabetes (10,397) OR (95% *CI*) ^b^	*P*	*P* trend	Excluding hypertension (8443) OR (95% *CI*) ^c^	*P*	*P* trend
Age (year)	variable						
[35–55]	0	1(Ref)			1(Ref)		
	[1–5)	1.21 (1.08–1.36)	0.01		1.21 (1.07–1.37)	< 0.01	
	[5–10]	1.52 (1.32–1.74)	< 0.01	< 0.01	1.49 (1.29–1.72)	< 0.01	< 0.01
[35–40)	0	1(Ref)			1(Ref)		
	[1–5)	1.17 (0.90–1.54)	0.25		1.00 (0.75–1.35)	0.98	
	[5–10]	1.17 (0.82–1.67)	0.39	0.32	1.00 (0.68–1.49)	0.96	0.96
[40–45)	0	1(Ref)			1(Ref)		
	[1–5)	1.28 (0.99–1.67)	0.05		1.24 (0.94–1.62)	0.12	
	[5–10]	1.57 (1.17–2.12)	0.03	0.03	1.32 (0.95–1.83)	0.09	0.08
[45–50)	0	1(Ref)			1(Ref)		
	[1–5)	1.14 (0.93–1.41)	0.20		1.19 (0.95–1.49)	0.13	
	[5–10]	1.39 (1.10–1.77)	< 0.01	< 0.01	1.45 (1.13–1.88)	0.04	< 0.01
[50–55]	0	1(Ref)			1(Ref)		
	[1–5)	1.05 (0.84–1.32)	0.68		1.13 (0.89–1.45)	0.32	
	[5–10]	1.40 (1.07–1.82)	< 0.01	0.01	1.49 (1.13–1.97)	< 0.01	< 0.01

^b^adjust age, gender, systolic blood pressure, high-density lipoprotein cholesterol, waist circumference, physical exercise, smoking, alcohol consumption, diabetes, baseline low-density lipoprotein cholesterol;

^c^adjust age, gender, systolic blood pressure, high-density lipoprotein cholesterol, waist circumference, physical exercise, anti-hypertensive treatment, smoking, alcohol consumption, baseline low-density lipoprotein cholesterol

## Discussion

The main finding of the present study was that prolonged exposure to hyperlipidemia in young adults increased the risk of arterial stiffness, especially in subjects aged 40–55 years who had been exposed to hyperlipidemia for > 5 years. After adjusting for confounding factors, the risk of hyperlipidemia in the high exposure and low exposure groups was significantly increased (1.22 and 1.49 times, respectively) compared with the normal group, and the trend was statistically significant. In subjects aged 40–55 years, the high exposure group had a significantly higher risk of arterial stiffness than the normal group, whereas there was no increased risk in the low exposure group. Thus, the risk of arterial stiffness increased with the duration of hyperlipidemia exposure in a dose-dependent manner, especially in individuals aged 40–55 years. Interestingly, there were no such differences in subjects aged 35–40 years, which may relate to the low incidence of arterial stiffness in this age group, and the small number of subjects exposed to hyperlipidemia for > 5 years. Although no previous studies have examined the relationship between prolonged hyperlipidemia exposure and arterial stiffness, Navar-Boggan [21] performed an average of 15 years of follow-up in 1478 young adults aged 35–55 years, and found that the risk of coronary heart disease increased 1.40 times every 10 years in 55-year-olds who had been exposed to non-high-density lipids. A meta-analysis of 312,321 subjects also reported that long-term exposure to naturally low levels of LDL-C was associated with a 54.5% reduction in the risk of coronary heart disease for each mmol/L lower LDL-C [10].

After adjusting for confounders, we also found that the effect of the duration of hyperlipidemia exposure on arterial stiffness was slightly decreased in subjects that did not meet statin treatment criteria under guidelines compared with the general population, although this remained a risk factor for arterial stiffness. Thus, it is understandable that the risk of arterial stiffness is slightly higher in young adults who are using drugs to treat hyperlipidemia according to current guidelines. However, most young adults with hyperlipidemia who are at risk of arterial stiffness do not meet statin treatment criteria under guidelines. Although no previous studies have examined whether early statin interventions can alleviate hyperlipidemia, and thus reduce the risk of arterial stiffness, in populations with prolonged exposure to low LDL-C, early use of statin was reported to be superior to late statin use, with each 1-unit decrease of LDL-C being associated with a three times lower risk of coronary heart disease [10]. In addition, the timing of treatment initiation in hyperlipidemic young adults at risk of arterial stiffness remains unclear. Unfortunately, our current research was also unable to answer these questions.

Because either diabetes or use of antihypertensive drugs may affect arterial stiffness, subjects with diabetes and hypertension were separately ruled out in our sensitivity analysis. We found that the relationship between prolonged exposure to hyperlipidemia and arterial stiffness in the different age groups did not change after the diabetes subjects were excluded. Furthermore, after the hypertensive subjects were excluded, prolonged exposure to hyperlipidemia showed no significant correlation with arterial stiffness in the 40–45-year-old group, and there were no changes in the other results. This may be because hypertension has a greater effect on arterial stiffness than on hyperlipidemia in the 40–45-year-old population, while the significance observed in the population without excluding hypertension might relate to the effect of hypertension on arterial stiffness.

The possible explanations for the findings in the current study are as follows. First, LDL-C is known to be harmful to endothelial function, and an elevated serum LDL-C level leads to arterial stiffness by increasing the vascular smooth muscle cell response to angiotensin II and reducing nitric oxide bioavailability [22, 23]. Second, the oxidized lipids accumulate along with the inflammatory reaction and migrate to the tunica intima, causing degradation of collagen, elastic fibers and proliferation of smooth muscle cells, thus leading to the development of arterial stiffness [24, 25].

There are a number of strengths of our study. First, we made full use of the continuous follow-up data concerning the 10-year period of potential hyperlipidemia exposure, and we accounted for arterial stiffness that preceded the cardiovascular events in the observed population. Considering that the association of the duration of hyperlipidemia with the risk of arterial stiffness may be age-related, we conducted an age-stratified analysis. Second, our study provides valuable information for both public health and clinical practice, as well as epidemiological evidence for future revisions of the guidelines on the prevention of dyslipidemia in adults. Clinicians should consider lifestyle interventions or statin therapy for adults with prolonged exposure to hyperlipidemia.

There are also some limitations of our study. The research population was limited to Kailuan Group employees, most of whom lived in communities in North China. Thus, the findings of this study may not be applicable to other populations. Nevertheless, the homogeneity of our cohort reduced the potential confounders, and the large sample size of the study was highly instructive for Chinese population. Another limitation is that we only considered the duration of hyperlipidemia exposure, without considering the duration of risk factors and complications. Indeed, exposure to certain complications can also affect the risk of arterial stiffness in a similar time-dependent manner. Nevertheless, we performed sensitivity analysis after hypertension and diabetes were excluded, and found that the relationship between the duration of hyperlipidemia exposure and arterial stiffness did not markedly change in the different age groups. In our cross-sectional study, we also did not detect arterial stiffness in the observed population, and were unable to rule out patients with arteriosclerosis. Thus, the strength of evidence was relatively weak. Furthermore, the Kailuan Study started in 2006, and prior LDL-C levels of the subjects were unknown. Thus, the exact duration of hyperlipidemia exposure is unknown. Nevertheless, our data provide LDL-C levels in the study population over 10 years, which is valuable for assessing the relationship between prolonged hyperlipidemia exposure and arterial stiffness. Finally, we used BaPWV for the arterial stiffness index, although carotid–femoral pulse wave velocity is the gold measure of central arterial stiffness. Nevertheless, previous studies have shown that BaPWV can be used to assess the degree of arterial stiffness in a simple, reproducible, and non-invasive way. Moreover, there is a strong correlation of BaPWV with carotid-femoral pulse wave velocity (correlation coefficient: 0.73). Therefore, BaPWV can be used to measure arterial stiffness [26].

## Conclusions

Prolonged exposure to hyperlipidemia in young adults increases the risk of arterial stiffness. Young adults with this condition may benefit from more aggressive primary prevention.

## Data Availability

Data sets that were generated and analyzed in this study will not be published because of data protection, but the appropriate authors may have access to and/or analyze the datasets from the current study if reasonably required.

## Abbreviations

LDL-C: Low-density lipoprotein cholesterol
BaPWV: Brachial-ankle pulse wave velocity
BP: Blood pressure
SBP: Systolic blood pressure
